# Quality of outpatient ambulatory surgical care: a systematic review and meta-analysis

**DOI:** 10.1186/s12893-026-03737-y

**Published:** 2026-04-21

**Authors:** Pooja Arumugam, Claire Fraser, Lisa Cassaniti, Joshua Wright, Sunnya Khawaja, Trang Dang, Alka Kothari, Manju Chandrasegaram, Isuru Ranasinghe

**Affiliations:** 1 Medical School, Faculty of Health, Medicine and Behavioural Sciences, The University of Queensland, Brisbane, QLD 4072 Australia; 2 Department of Cardiology, The Prince Charles Hospital, Brisbane, 4032 Australia; 3 Department of Obstetrics and Gynaecology, Redcliffe Hospital, Redcliffe, 4020 Australia; 4 Department of Surgery, The Prince Charles Hospital, Brisbane, 4032 Australia

**Keywords:** Ambulatory surgery, Surgical quality, Healthcare outcomes, Adverse events, Inpatient hospitalisation

## Abstract

**Background:**

Most surgical procedures in Australia are performed in the ambulatory (outpatient) setting; however, the quality of ambulatory surgical care is still poorly understood. Existing studies typically report outcomes for individual ambulatory surgical procedures, yet this limited scope does not capture the overall quality across the diverse range of ambulatory surgical procedures. We conducted a systematic review and meta-analysis to examine the safety and quality of ambulatory surgery, focusing on the facility-wide incidence of adverse clinical outcomes that occur within 30 days of surgery.

**Method:**

We searched PubMed and Embase for studies published between January 2000 and July 2023, including studies reporting outcomes within 30 days following facility-wide ambulatory surgeries. Pooled estimates were calculated using the Freeman-Tukey double arcsine transformation. Publication bias was assessed through visual examination of funnel plots and Egger’s test for funnel plot asymmetry.

**Results:**

From 4,298 records screened, we identified 30 studies encompassing 15,868,214 patients. Immediate hospitalisation was reported commonly (12 studies, pooled proportion 1.82%, 95% CI 1.1–2.71%), and 30-day hospitalisation was reported by nine studies (pooled proportion 2.88%, 95% CI 1.2–5.3%). Death and post-operative complications were infrequently reported. The most commonly reported causes of hospitalisations were surgical (34.4% of all hospitalisations, 95% CI 31.2–37.7%), organisational (26.0%, 95% CI 12.5–42.2%), and anaesthetic concerns (11.8%, 95% CI 9.6–14.2%). All pooled results showed a high degree of heterogeneity, reflecting differences in surgical cohorts and outcome definitions.

**Conclusions:**

Although ambulatory surgery appears to be associated with relatively low rates of conversion to inpatient hospitalisation or 30-day hospitalisation, these findings need to be interpreted with caution due to the heterogeneity in cohort and outcome definitions.

**Supplementary Information:**

The online version contains supplementary material available at 10.1186/s12893-026-03737-y.

## Background

In many developed countries such as the United States, Europe and Australia, over 70% of all surgical procedures are performed as ambulatory (outpatient) procedures, often at specialised facilities such as ambulatory or day surgical centres (ASCs) [1, 2]. The rapid growth in ambulatory surgery is primarily driven by advancements in anaesthesia and the development of minimally invasive techniques, such as laparoscopic approaches, which enable quicker recovery times, eliminate the need for hospital admission, and reduce costs for patients [3]. For hospitals and health services, ambulatory surgery frees up inpatient beds for individuals requiring more complex or urgent care. Additionally, ambulatory surgery generally incurs lower costs than inpatient surgery because it requires fewer hospital resources and avoids overnight stays [3], thus providing strong incentives for adoption. Indeed, reflecting this shift, there are now more than 6,000 ASCs in the United States alone [4].

As ambulatory care settings expand to include more medically complex patients and procedures, concerns regarding the quality of ambulatory surgical care are growing. However, the overall quality of ambulatory surgical care remains poorly understood. Compared with inpatient surgery, ambulatory settings have been less thoroughly researched. Existing studies typically report outcomes for individual procedures in ambulatory care [5–12], yet this limited scope does not capture the overall quality of care across a diverse range of procedures performed in these settings. Moreover, evidence suggests that some ambulatory facilities lack essential equipment for monitoring complex cases or handling medical emergencies [13]. Furthermore, quality reporting initiatives are far less stringent in ambulatory settings than in inpatient care, leaving considerable uncertainty regarding the safety, quality, and outcomes of ambulatory surgery. Addressing this knowledge gap is critical for clinicians, health services, and policymakers who require reliable data to develop strategies for monitoring and enhancing care within ambulatory settings.

Accordingly, we conducted a systematic review and meta-analysis to assess the rates, timing, and causes of adverse outcomes after contemporary ambulatory surgery, to evaluate the overall quality of ambulatory surgery. Our analysis focused on studies that comprehensively reported the outcomes across a wide array of procedures in ambulatory settings. We restricted our review to outcomes occurring within 30 days of surgery to identify those most directly related to ambulatory procedures. Prior research has shown that most adverse outcomes associated with ambulatory surgery manifest within this time frame [14, 15].

## Method

We followed the Preferred Reporting Items for Systematic Reviews and Meta-Analysis (PRISMA) guidelines (See Supplementary Tables S5 and S6) [16].

### Search strategy

The PubMed and Embase search strategies were designed in consultation with a librarian to identify eligible studies published between 2000 and 2023 (see Supplementary Table S1 for the search queries used). Primary research articles reporting outcomes within 30 days following facility-wide ambulatory surgeries in adult patients aged over 18 years were included.

The exclusion criteria were as follows: (i) studies with sample size less than 100; (ii) studies focusing on outcomes of a single ambulatory surgical procedure or a single surgical specialty; (iii) studies that focused on procedures using a single type of anaesthetic protocol; (iv) studies that reported outcomes beyond 30 days following surgery; (v) studies that focused on a specific subgroup of patients (specific comorbidity, ethnicity, sex, etc.); (vi) studies including paediatric patients aged less than 18 years; (vii) studies comparing or testing specific interventions; (viii) studies such as reviews, case studies, abstracts, letters, editorials, and interventions; (ix) studies that were published after 2000 but included study data prior to 2000. Studies were also excluded if they were animal studies, non-English studies, studies without available full text, or if they included the same surgical cohort as another included study.

After removing duplicates, three investigators (PA, LC, and CF) reviewed the abstracts of the identified publications for inclusion. This was followed by a full-text review, and discrepancies were resolved through group discussion to reach a consensus. PA reviewed all the studies, whereas LC and CF reviewed half of the studies to ensure that each study was assessed for inclusion by two authors. The references of the included articles were screened to identify additional eligible publications.

### Quality assessment

Two investigators (PA and JW) independently assessed the quality of each included publication using the National Institute of Health Quality Assessment Tool [17], which consists of 14 criteria for evaluating the quality of cohort studies. Discrepancies were resolved through consensus. Studies were rated as ‘Good’ if all criteria were met with a ‘Yes’ or ‘NA’ response, ‘Fair’ if up to two criteria received a ‘No’ response, and ‘Poor’ if three or more criteria were rated as ‘No.’

### Study outcomes

We evaluated four outcomes relevant to the quality of ambulatory care. These included (1) conversion to inpatient hospitalisation, defined as an unplanned inpatient hospital admission on the day of surgery without discharge, or hospital stay duration greater than 24 h; (2) unplanned hospitalisations following ambulatory surgery, defined as any unplanned hospital admission or emergency department (ED) visit within 30 days of surgery, not including immediate conversions to inpatient hospitalisation; (3) complications of ambulatory surgery, defined as any complication occurring at the time of surgery or in the post-operative follow-up period up to 30 days, irrespective of whether the complication required hospitalisation; (4) mortality within 30 days of surgery. We also examined the reasons for hospitalisation and patient- and procedure- related risk factors for the reported outcomes.

### Data extraction

Data from the included studies were extracted using a standardised data extraction form. The proportions of hospitalisations and complications were extracted from figures or text or calculated from the number of patients. Risk factors for the outcomes were extracted from the text. Extraction was performed independently by two investigators (PA and JW) to minimise extraction errors, and discrepancies were resolved by consensus.

### Statistical analysis

Data for categorical variables were summarised as frequencies and percentages. Continuous variables were presented as mean ± standard deviation or median and interquartile range. All unadjusted quantitative outcomes were recorded as the number and proportion of patients who underwent surgery.

The results of the meta-analysis of continuous variables are reported as the pooled mean and 95% confidence interval (CI). The meta-analysis of proportions was performed using a Freeman–Tukey double arcsine transformation, with results reported as the pooled proportion and 95% CI. Owing to potential differences between studies, we used a random-effects model to calculate the pooled outcomes. The I^2^ statistic was used to evaluate the heterogeneity among studies, and the between-study variance τ2 was calculated using the restricted maximum likelihood estimator [18].

### Risk of bias assessment and subgroup analysis

Publication bias was evaluated through visual examination of funnel plots, and Egger’s test was used to assess for the presence of plot asymmetry [19]. Where Egger’s test indicated possible funnel plot asymmetry, a trim and fill analysis was performed to evaluate the change in pooled estimate when plot asymmetry was accounted for. This analysis works by removing studies with extreme estimates and filling in missing imputed studies based on bias-corrected estimates [20]. Subgroup analysis was performed by study design (prospective vs. retrospective), study quality (good vs. fair vs. poor) and data source (hospital vs. a national database).

### Sensitivity analysis

To evaluate whether the pooled estimates of proportions were affected by the back transformation method, we performed a sensitivity analysis by repeating the meta-analysis of proportions of outcomes using a logit transformation [21]. All analyses were performed using the ‘metafor’ package in R [22], with a two-tailed p-value of < 0.05 considered statistically significant.

## Results

**Fig. 1:**
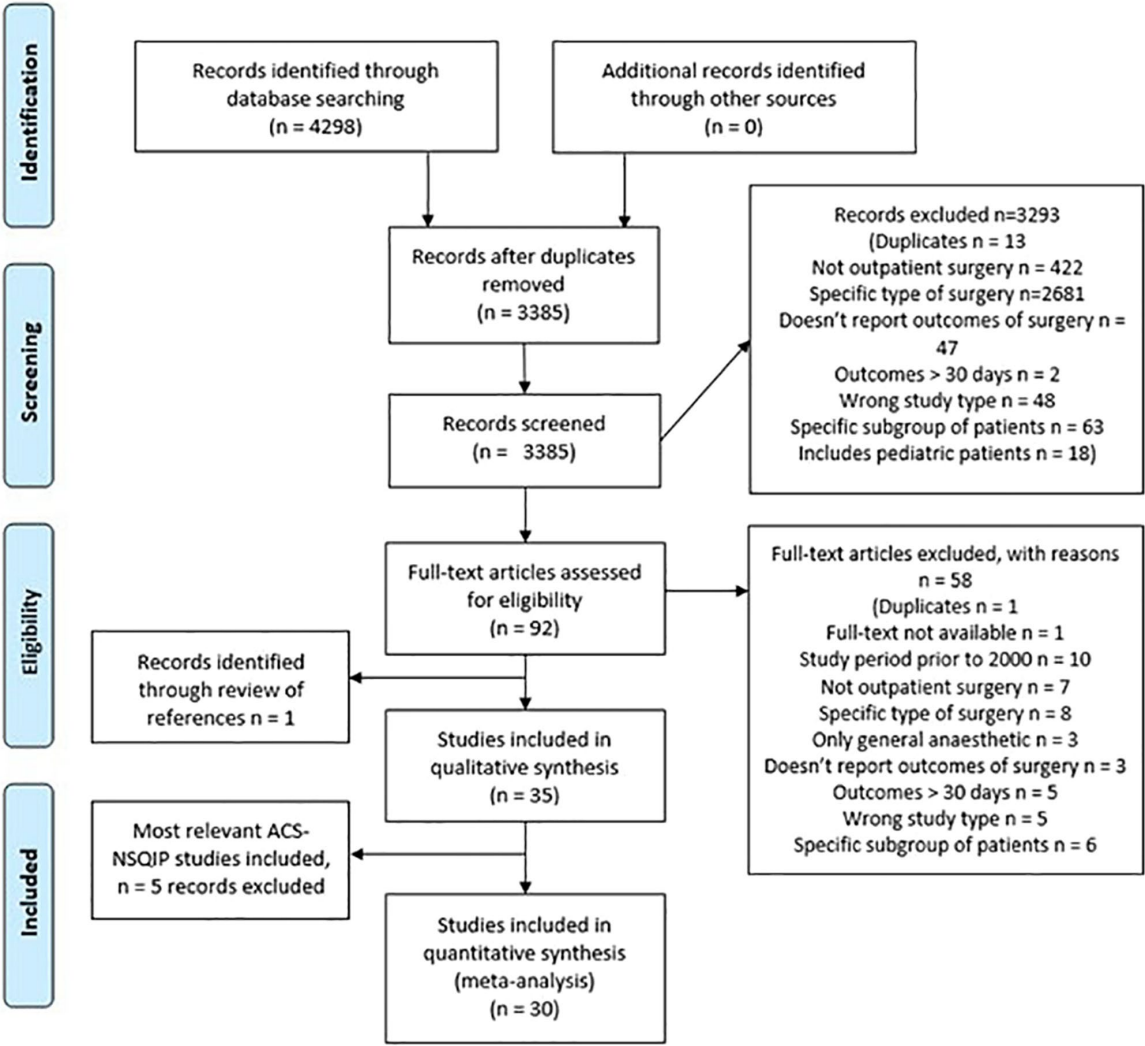
PRISMA study selection flow diagram

We identified 4,298 studies published between 1 st January 2000 and 31 st July 2023. After removing duplicates and applying the exclusion criteria, 30 studies [23–52] encompassing 15,868,214 patients were included in the review (Fig. 1).

### Study characteristics

The characteristics of the included studies are summarised in Table 1. Most studies were retrospective (66.7%) or prospective cohort studies (20%). Most studies (66.7%) were good, 30% were fair, and 3.3% were of poor quality. Half (50%) of the studies originated from the United States (US), 10% from France, 10% from the United Kingdom, and 6.7% from Canada. The remaining studies originated from either other European countries, China, or one study that included data from multiple countries (Table 1).

**Table 1:** Characteristics of included studies

Characteristics	Number of studies (number of patients)	Summary estimate (95% CI) (%)
Study Design		
Prospective cohort	6 (3834)	20.0
Retrospective cohort	20 (15504658)	66.7
Retrospective - Other	2 (74427)	6.7
Prospective - Other	2 (1197)	6.7
Data Source		
Single Centre	16(1460841)	53.3
Multicentre/national registries	14(14122731)	46.7
Country		
US	15 (15030635)	50.0
UK	3 (2129)	10.0
France	3 (41569)	10.0
Canada	2 (781167)	6.7
Other (Spain, Netherlands, Belgium, Ireland, Italy, China, International)	7 (12714)	23.0
Quality Assessment		
Good	20 (15533195)	66.7
Fair	9 (79756)	30.0
Poor	1 (602)	3.3
Patient Demographics		
Age (years, mean)	12 (3206209)	53.3 (49.1–57.5)
Male (%)	26 (15218624)	48.8 (40.3–57.4)
ASA I (%)	9 (5970588)	37 (21.2–54.4)
ASA II (%)	9 (5970588)	44.2 (33.7–55.1)
ASA III (%)	9 (5970588)	19.5 (11.8–28.7)
Smoker (%)	7 (7332055)	10.6 (4.5–18.)
Type of Surgery		
General	15 (8668320)	25.8 (16.1–36.9)
GIT Endoscopy	5 (4575311)	39.4 (28.3–51.1)
Neurological	6 (9734161	6.56 (5.0–8.3.0.3)
Vascular	6 (8122484)	4.4 (1.2–9.6)
Orthopaedic	21 (11423107)	18.1 (12.8–24.2)
Thoracic	(3) 2,897,216	1.2 (0.3–3.7)
Respiratory	4 (2820640)	1.2 (0.3–2.6)
Otolaryngology	(11) 4,199,567	4.6 (2.8–6.7)
Urology	(16) 10,928,862	7.6 (3.7–12.6)
Plastic	(6) 2,932,258	5.3 (3.3–7.8)
Digestive	(5) 2,821,184	19.9 (8.4–34.9)
Gynaecology	(11) 7,487,441	8.4 (3.6–11.4)
Skin	5 (6358912)	7.1 (3.7–11.4)
Ophthalmology	(10) 6,056,327	13.9 (8.2–20.7)
Breast	6 (4151952)	9.9 (3.6–19.0)
Comorbidities (%)		
Diabetes	7 (8256107)	9.2 (3.2–17.8)
Hypertension	8 (8467496)	32.0 (18.5–47.4)
Heart Failure	5 (8255019)	3.6 (0.1–11.2)
Coronary Artery Disease	3 (1685199)	3.2 (0.5–8.2)
Disseminated Cancer	2 (5645168)	0.5 (0.5–0.5)
COPD	7 (7332055)	6.2 (0.7–16.8)
Dialysis	2 (5645168)	0.6 (0.6, 0.6)
Renal Failure	4 (4507988)	0.8 (0.0–2.7)
Obesity	6 (6993686)	27.3 (15.9–40.6)
Asthma	2 (70639)	18.0 (0, 60)
Bleeding Disorder	2 (5645168)	1.6 (1.6–1.6)
Dyslipidaemia	2 (1688)	26.11 (14.9, 39.29)

*US* United States, *UK* United Kingdom, *ASA* American Society of Anaesthesiologists, *GIT* Gastrointestinal tract, *COPD* Chronic obstructive pulmonary disease

The pooled mean age of the patients was 53.3 ± 4.2 years, and 48.8% were male. The patients were selected from individual hospitals (53.3%) or nationwide databases (46.7%). Many patients had comorbid hypertension (pooled proportion 32%, 95% CI 18.5–47.4%), and there was also a high prevalence of obesity (27.3%, 95% CI 15.9–40.6%) and dyslipidaemia (pooled proportion 26.1%, 95% CI 14.9–39.2%). Other comorbidities were less prevalent (Table 1).

The common surgical procedures performed in the ambulatory setting that were included in the publications are outlined in Table 1. The most common procedures performed were as follows: general surgical procedures (pooled proportion 25.8%, 95% CI 16.1–36.9%), which included hernia repair, cholecystectomy, and haemorrhoid surgery; gastrointestinal endoscopic procedures (pooled proportion 39.4%, 95% CI 28.3–51.1%); digestive system surgery (pooled proportion 19.9%, 95% CI 8.4–34.9%), including liver, appendix, colorectal, and other abdominal surgeries; orthopaedic surgery (pooled proportion 18.1%, 95% CI 12.8–24.2%), which commonly included arthroscopy, ligament reconstruction, and joint replacement procedures; and ophthalmological procedures (pooled proportion 13.9%, 95% CI 8.2–20.7%), including cataract, corneal transplantation, and strabismus surgery. Breast, gynaecological, urological, and integumentary procedures were less common. Details of the individual studies and the results of the study quality assessment are provided in Supplementary Appendix Tables S2 and S3.

### Outcomes

#### Conversion to an inpatient hospitalisation

**Fig. 2:**
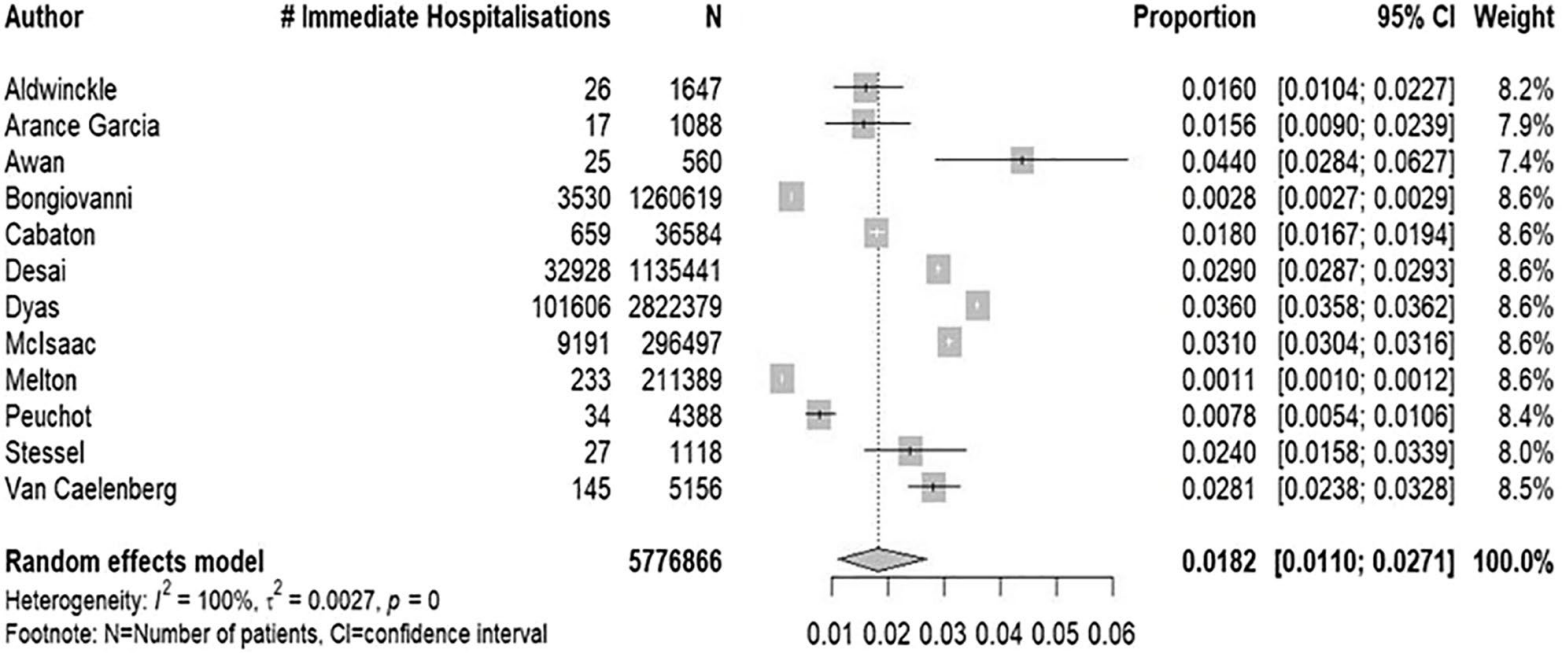
Forest plot of conversion to an inpatient hospitalisation following day surgery (Number of studies = 12, *N* = 576866)

The conversion of the outpatient day procedure to inpatient hospitalisation was the most common outcome reported in 12 publications (*n* = 5,776,866). The pooled proportion of procedures requiring conversion to inpatient hospitalisation was 1.8% (95% CI 1.1–2.7%). However, the risk of conversion reported in individual studies ranged from 0.1% to 4.4%, with extreme heterogeneity between the studies (I^2^ = 100, Fig. 2).

#### Hospitalisations within 30 days of surgery

**Fig. 3:**
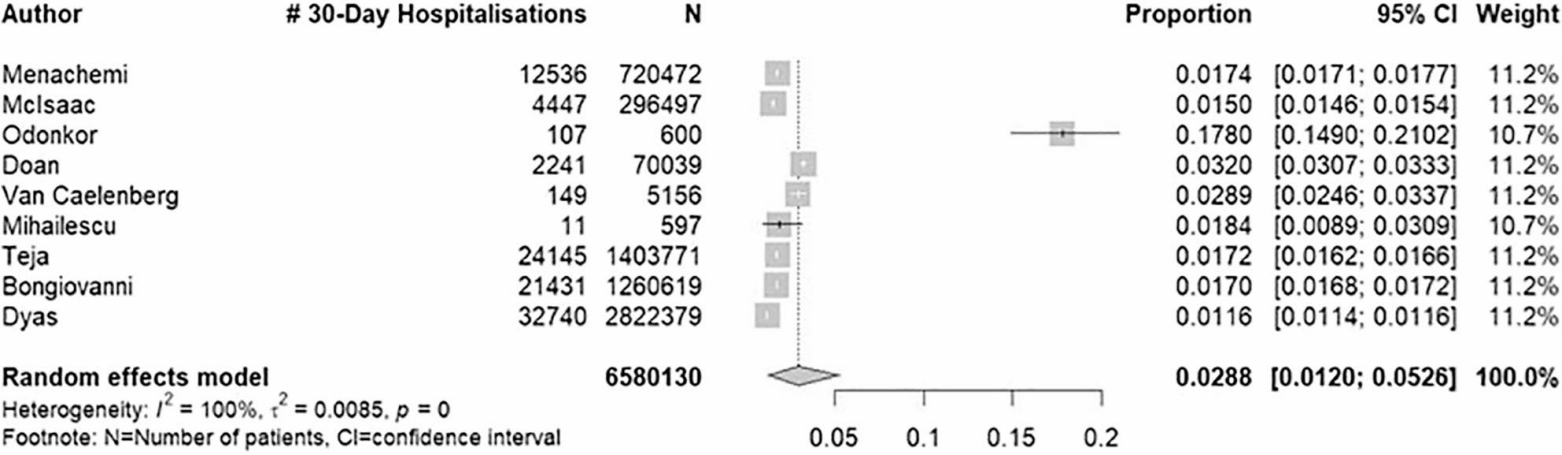
Forest plot of 30-day hospitalisation following day surgery (Number of studies = 9, *N* = 6580130)

Few studies have reported hospitalisation following discharge, with significant heterogeneity in the follow-up period among individual studies. Hospitalisation within 30 days of surgery was the most common outcome timeframe and was reported in nine studies. The pooled proportion of 30-day hospitalisations (excluding conversion to inpatient hospitalisation) across these nine studies was 2.9% (95% CI 1.2–5.3%, I^2^ = 100, Fig. 3). Three studies also reported hospitalisations within seven days of discharge [39, 41, 42]. A meta-analysis of these was not performed due to the insufficient number of studies, but the reported hospitalisation rate varied from 0.5% to 16%.

#### Reasons for hospitalisations

The reasons for conversion to inpatient hospitalisation were reported in seven publications encompassing 3,083,041 patients. Diverse terminologies were used to describe and categorise the reasons for these hospitalisations, with some publications categorising reasons into surgical, medical, anaesthetic, organisational, and social factors, while others reported the frequency of individual reasons for inpatient hospitalisations, such as bleeding and mobility issues.

**Fig. 4:**
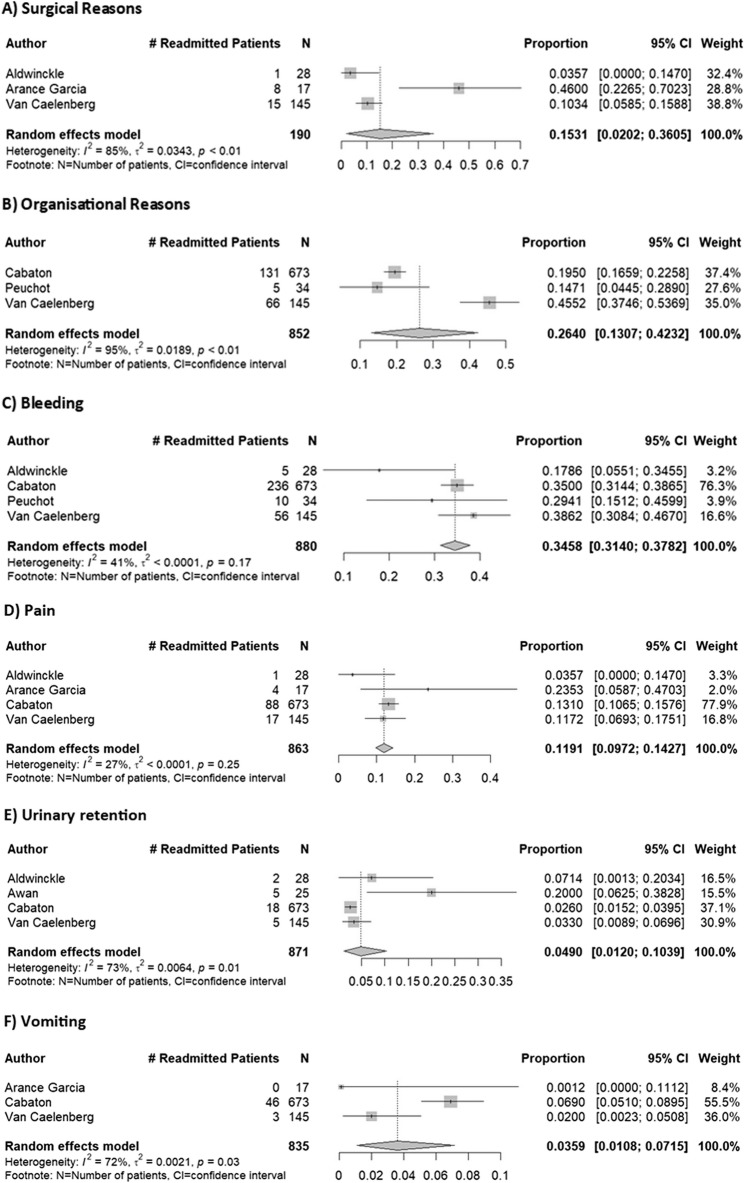
Forest plots of reasons for readmission following day surgery

Among the studies that broadly categorised reasons for conversion to inpatient hospitalisation, four discussed surgical reasons [24, 28, 46, 52]. The pooled proportion reported was 34.5% of all hospitalisations (95% CI 31.4–37.8%), with moderate heterogeneity (I^2^ = 40.6%, Fig. 4**).** Three reported organizational reasons, such as the delayed start of the procedure and late exit from the theatre [28, 46, 52]. These accounted for a pooled proportion of 26.4% (95% CI 13.1–42.3%) of hospitalisations, with high heterogeneity (I^2^ = 95%). Medical and anaesthetic factors were only reported in two studies; therefore, they were not pooled. However, the proportion converted to inpatient hospitalisations varied from 5.5–7.1% [24, 52] due to medical and 10.3–56% [46, 52] due to anaesthetic reasons. Social reasons for hospitalisation were also reported in two studies, accounting for 10.5–10.7% of hospitalised patients [24, 28].

Of the studies that reported individual reasons for conversion to inpatient hospitalisation (Fig. 4), the most common surgical reasons included bleeding (pooled proportion 15.3%, 95% CI 2–36.1.1%, I^2^ = 84.6% across three studies [24, 25, 52] and postoperative urinary retention (pooled proportion 4.9%, 95% CI 1.2–10.4%, I^2^ = 73.5% across four studies [24, 25, 28, 52). Reoperation was reported in only two publications [25, 52] and accounted for 1.4–5.9% of hospitalisations. The most common anaesthetic reasons were pain (pooled proportion 11.9%, 95% CI 9.7–14.3%, I^2^= 27.1%) across four studies [24, 25, 28, 52], and vomiting (pooled proportion 3.6% (95% CI 1.1–7.2%, I^2^= 71.7%) across three studies [25, 28, 52]. Individual medical reasons for conversion to inpatient hospitalisation have not been widely reported. However, in one publication, mobility issues were cited as a reason for hospitalisation in 21.4% of the readmitted patients [24].

#### Risk factors for re-hospitalisations

Many studies have also evaluated patient or procedural risk factors associated with an increased risk of hospitalisation or complications following surgery (Table 2). Ten reported risk factors for conversion to inpatient hospitalisation, and eight studies reported risk factors for 30-day hospitalisation. The risk factors for both outcomes were similar and included increasing age (particularly over 60 years) [24, 27, 28, 30, 31, 38, 43, 49], higher ASA class [24, 28, 31, 35, 38, 49], low socioeconomic status [43], male sex [26, 30], previous hospitalisation [44], and an increased number of comorbidities such as cardiovascular disease, obesity, COPD, and bleeding disorders. [23, 25, 27, 30, 31, 36, 43, 46, 51] A number of procedural factors were also cited, notably prolonged surgery duration, use of general anaesthesia, and surgery being performed in hospital outpatient departments (HOPDs) as compared to ASCs [23, 26–28, 31, 35, 38, 43, 47, 49, 51, 52].

**Table 2:** Risk factors for hospitalisation

Risk Factor	Number of studies describing risk factor for conversion to inpatient hospitalisation		Number of studies describing risk factor for 30-day hospitalisation		Explanation of risk factor
Procedural Factors	6 [26, 28, 35, 38, 49, 52]	5 [23, 27, 39, 43, 51]		Several procedural factors were associated with higher risk of readmission: -Prolonged surgery duration -Use of general compared to local anaesthetic -Time of completion of surgery later in the day -Surgery being performed at HOPD -Certain surgical specialities	
Comorbidities	2 [25, 28]	5 [23, 27, 36, 43, 51]		Increasing comorbidities were associated with higher risk of readmissions, notably: - Cardiovascular disease, including heart failure, ischemic heart disease, diabetes, obesity -Bleeding disorder -COPD -Disseminated cancer -Obesity Depression Renal Failure	
Age	4 [26, 28, 38, 49]	5 [23, 27, 30, 31, 43]		An increase in age was associated with increased risk of readmissions, specifically age over 50–60 years	
ASA Category	4 [24, 28, 38, 52]	1 [23]		Increased ASA class was associated with higher risk of readmission, particularly ASA class of 3 or higher	
SES	0	5 [43]		Lower SES was associated with higher risk of readmission, including: -Rural living -Decreased social support -Lesser income -Being unmarried -Minority race -Living increased distance from hospital	
Sex	1 [26]	1 [30]		Being male increased the risk of readmission following surgery	
Previous Hospitalisation	2 [44]	0		Previous hospitalisations increased the risk of readmission, particularly previous hospitalisation for surgery	

*HOPD* Hospital-based outpatient department, *COPD* Chronic obstructive pulmonary disease, *ASA* American Society of Anaesthesiologists, *SES* Socioeconomic status

#### Postoperative complications

Postoperative complications were defined as any complications that occurred during the postoperative period, irrespective of whether they led to hospitalisation. Most studies reported complications immediately following surgery (within 24 h). Table 3 summarizes the postoperative complication rates. However, post-discharge complications are rarely reported.

**Table 3:** Postoperative complications (within 24 h)

Postoperative complication	Immediate complications	
	Number of Studies (number of patients)	Proportion of Patients (%)
Urinary Retention [47, 52] (%)	2 (984)	4.2–15.7
Pain [27, 28, 47, 49] (%)	4 (7725)	1.0–73
Surgical site infection [39] (%)	1(354121)	0.63
Nausea [27] (%)	1(1647)	1.2
Vomiting [27, 28, 47, 49] (%)	4 (7725)	0–27.7.7
Fever [28] (%)	1(1088)	0.64
UTI [39] (%)	1(354121)	0.45
Dizziness [27, 28] (%)	2 (2735)	0.4–1.5
Cardiac [39] (%)	1(354121)	0.14
Stroke [31] (%)	1(354121)	0.05
Bleeding [27, 28, 34, 39, 47] (%)	5 (3179837)	0.0–10.8.0.8
Cardiac arrest [39, 41] (%)	2 (565510)	0.0–0.08.0.08

*UTI* Urinary tract infection

**Fig. 5:**
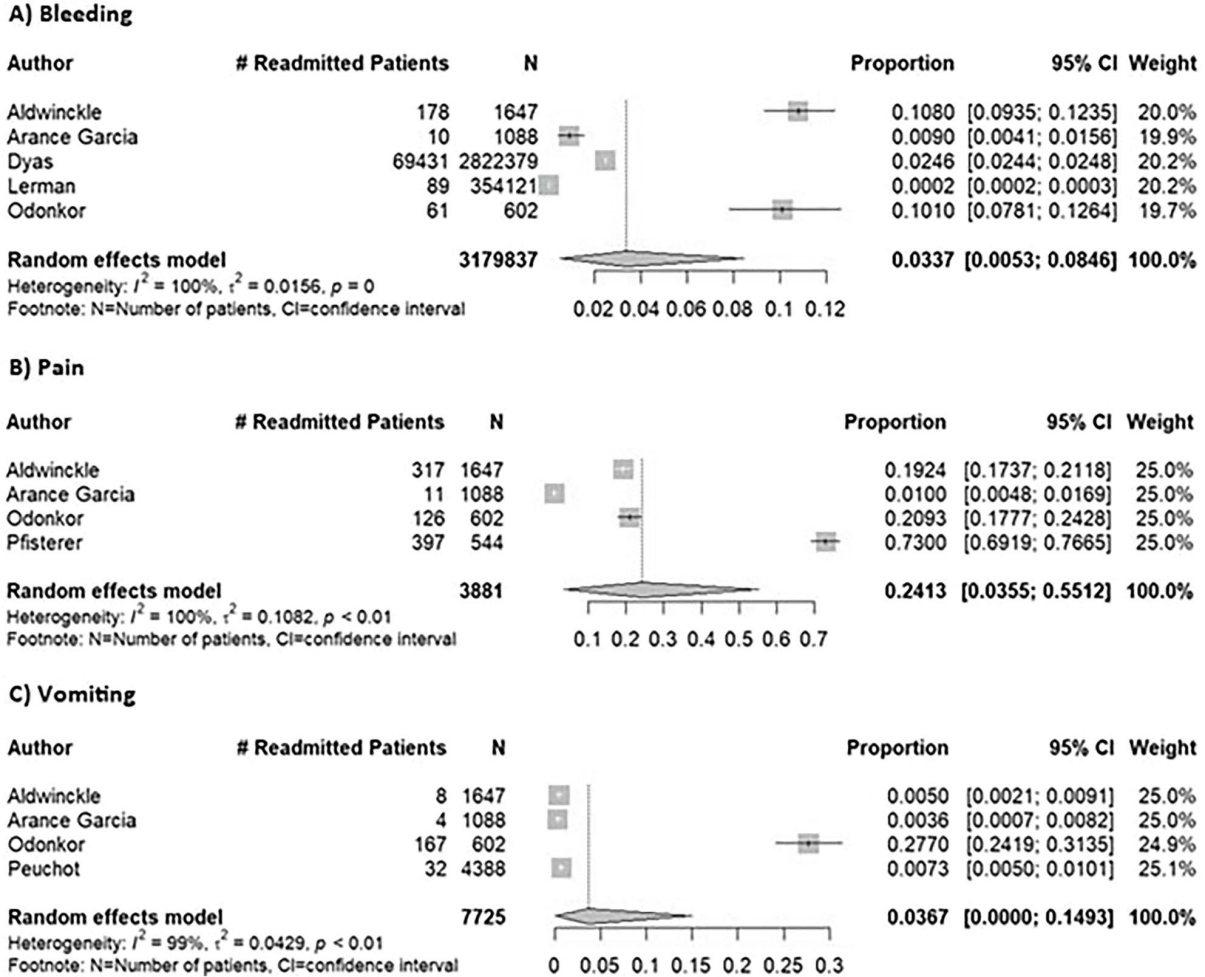
Forest plots of postoperative complications following day surgery

The most common postoperative complications are pain, vomiting, and bleeding. Postoperative pain was reported in four studies, with a pooled proportion of 24.13% of all patients (95% CI 3.55–55.12%) and high heterogeneity (I^2^= 99.8%) [24, 25, 44, 46]. Vomiting had a pooled proportion of 3.67% (95% CI 0–14.93.93%) and high heterogeneity (I^2^ = 99.4%) across four studies [24, 25, 44, 46]. Bleeding had a pooled proportion of 3.37% (95% CI 0.53–8.46%) and high heterogeneity (I^2^ = 100%) across five studies [24, 25, 31, 36, 45] **(**Fig. 5**)**. Urinary retention was also reported in 4.2–15.7% of patients; however, it was only reported in two studies [44, 49]. Other complications, including surgical site infection, nausea, and urinary tract infection, were reported at rates of less than 2% [36]. There was significant heterogeneity between publications in the reported rates of these complications, even when the same follow-up period was used.

#### 30-day mortality

Only two publications reported 30-day mortality with an extremely low rate of 0.05–0.06% incidence of death [23, 37].

### Publication bias

Funnel plots and the results of Egger’s test to determine the risk of publication bias are presented in Supplementary Appendix Figures S1-S2. Egger’s test did not indicate the presence of funnel plot asymmetry in the meta-analysis of the incidence of conversion to inpatient hospitalisation (*p* = 0.61). Similarly, Egger’s test did not indicate funnel plot asymmetry in the analysis of 30-day hospitalisations (*p* = 0.07). However, as the number of studies reporting this outcome was less than 10, the use of Egger’s test to determine publication bias may be unreliable.

### Sensitivity or subgroup analysis

Subgroup analysis was only performed for the outcome of conversion to inpatient hospitalisation because of the limited number of studies that reported other outcomes. The reported incidence of conversion to inpatient hospitalisation was similar between good- and fair-quality studies (1.81% vs. 1.86%, *p* = 0.94) and between studies using individual hospital data and those derived from national databases (2.0% vs. 1.56%, *p* = 0.6). The incidence of conversion to inpatient hospitalisation was higher in prospective studies than in retrospective studies (3.61% vs. 1.6%, *p* = 0.035), although only two studies were included in the prospective subgroup. There were no significant differences when calculating the pooled risk of conversion to inpatient hospitalisation (including subgroup analysis) using the logit transformation instead of the Freeman-Tukey double arcsine transformation (see Supplementary Table S4).

## Discussion

In this systematic review and meta-analysis, we evaluated the contemporary rates of adverse clinical outcomes occurring within 30 days of ambulatory surgery, focusing on publications published over the last two decades. Despite the widespread use of ambulatory surgery, comprehensive studies examining outcomes across the full range of procedures performed in this setting are lacking. Most of the included studies reported the incidence of conversion to inpatient hospitalisation, with a pooled estimate of 1.82%, which was primarily attributed to surgical complications, anaesthetic factors, and organisational issues, many of which may be preventable. However, post-discharge outcomes were reported less frequently. Hospitalisations within 30 days were the most commonly documented post-discharge outcome, with a pooled estimate of 2.88%. However, the underlying reasons for these hospitalisations, as well as the rates of complications and deaths following discharge, are rarely provided, leaving significant gaps in understanding post-discharge outcomes. Furthermore, the data did not allow for reliable estimation of the risks associated with major surgical types due to limited reporting. These results also underscore the need for caution when interpreting pooled estimates owing to the substantial heterogeneity in cohort characteristics and outcome definitions. Collectively, these findings highlight critical gaps in the understanding of the safety and quality of ambulatory surgery and have important implications for clinical and policy efforts to improve care quality in this setting.

To our knowledge, this is the first systematic review and meta-analysis on the incidence of clinical outcomes following a wide range of ambulatory surgical procedures. Prior reviews are limited to narrative reviews that have primarily focused on the risks and reasons for conversion to inpatient hospitalisation [53–57]. Our meta-analysis pooled 30 studies covering > 15,000,000 patients, allowing for a better understanding of adverse events that follow ambulatory surgery. We found that conversion to inpatient hospitalisation was low, although it most frequently occurred because of postoperative surgical or anaesthetic reasons. However, organisational factors, such as late start, delayed surgery time, and lack of an escort home, also contributed. Many of these factors are potentially preventable [58, 59] and thus provide an opportunity to further reduce the incidence of conversion to inpatient hospitalisation.

In contrast, post-discharge outcomes have been less frequently reported. Although we demonstrated that 2.88% of the patients were hospitalised within 30 days of discharge, this result must be interpreted with caution, as only nine studies reported this outcome with significant heterogeneity. Indeed, rehospitalisation rates of up to 10% have been reported at 7 days in large-scale population studies in the USA with Medicare patients older than 65 years [29]. These patients may represent an older, sicker cohort eligible for Medicare benefits, which may explain the higher rehospitalisation rates. Furthermore, they included visits to emergency departments and short-stay units in addition to inpatient hospitalisations, which may have contributed to the higher incidence rate. Post-discharge deaths and complication rates were also rarely reported. We also found limited data on the reasons for hospitalisation after discharge, indicating that the post-discharge outcomes of ambulatory surgery remain poorly understood. Although day surgery is generally considered safe, it is crucial to understand the adverse outcomes after discharge and the underlying factors that may drive these outcomes in order to inform patients, physicians, and quality improvement efforts. It is difficult to draw conclusions regarding the outcomes and adverse complications of ambulatory surgery beyond the immediate post-operative period. Further studies are required in this area.

Our study identifies additional knowledge gaps. The publications included in this systematic review did not permit reliable estimation of adverse outcomes associated with major surgical types or specialties performed in the ambulatory setting, such as abdominal and orthopaedic surgery. This was due to included studies rarely reporting adverse outcomes of individual surgical types or specialities, meaning sufficient data to calculate pooled estimates was not available. Consequently, although the average rate of conversion to inpatient hospitalisation or hospitalisation at 30 days was low, it was not possible to determine whether these rates were consistent among the surgical types. Similarly, we could not ascertain whether death and complications were similar across a broad array of surgical procedures. This is important because, in addition to patient factors, we found that surgical specialty and type of procedure impacted the rate of adverse outcomes [24–29, 31, 32, 37, 39, 40, 42–45]. Future studies should stratify the prevalence of adverse outcomes by type of surgery. This will enable a clearer understanding of the consistency of risk estimates across surgical subtypes, help identify particularly high-risk procedures and inform when surgery may be inappropriate in the outpatient setting for specific patient groups.

Many of the studies included in the meta-analysis were performed in the US, whereas the remaining studies were conducted in European countries. The composition of the surgical procedures performed and the quality of ambulatory surgery are likely to differ across countries and health systems, particularly in developing countries. Further research is required to determine how adverse outcome rates vary across the healthcare systems.

These findings have important implications for clinical and policy efforts aimed at improving ambulatory care. A large proportion of conversion to inpatient hospitalisations was due to potentially preventable surgical, anaesthetic, and organisational reasons, such as postoperative complications including pain and nausea, suggesting that quality measures targeting conversion to inpatient hospitalisation are useful. Implementing interventions aimed at reducing postoperative complications and rehospitalisation may improve care. For example, comprehensive surgical checklists markedly reduce complications and almost halve mortality [60–64]. Likewise, surgical care pathways provide better pain control, lower complication rates, and reduce postoperative length of stay by 28% [65]. Similarly, multidisciplinary discharge planning and transitional care interventions have been shown in randomised trials to reduce rehospitalisations by 30% in hospitalised patients [66]. Implementing similar measures in an ambulatory care setting offers an opportunity to reduce rehospitalisations and can be applied across a broad range of surgical specialties.

While existing studies have reported outcomes of ambulatory surgery in single specialities, understanding the facility-wide rates of adverse outcomes after ambulatory surgery is crucial in the context of increasing clinical and policy interest in understanding the overall quality of ambulatory surgical care. These data are important, as they allow for the development and implementation of quality metrics across this setting by informing the key outcomes reported in the literature to assess the quality of ambulatory surgical care. For example, current facility-wide quality measures include unplanned hospital visits within 7 days of ambulatory surgery, which is a publicly reported measure of ambulatory surgical quality among hospitals in the US [29, 67]. Furthermore, understanding factors that influence these outcomes is also useful to develop risk adjustment models for such measures and to guide targets for facility-wide quality improvement activities. For example, existing surgical safety checklists focus on implementing facility-wide quality improvement strategies such as orally confirming patient identity and surgical site, having a standardised plan for management of high-volume blood loss and standardised timeframes for administration of antibiotic prophylaxis [60]. However, these have not yet been extended into the outpatient setting. Understanding risk factors for adverse events in ambulatory surgery may assist in developing surgical safety checklists that can be implemented facility-wide in the ambulatory care setting.

Mortality and other major complications in ambulatory care settings have been described as poor indicators of care quality owing to their relatively low prevalence [54]. However, very few studies have reported the incidence of these outcomes, particularly after discharge. Therefore, mortality and major complications should not be assumed to be low and should be measured whenever feasible.

Certain limitations of this study should be noted when interpreting these results. There was a significant degree of heterogeneity among the included studies. The main sources for this heterogeneity were the differing data sources. Sixteen studies used single-centre data (mainly HOPDs), while 14 drew from multicentre or national registries of ambulatory surgery outcomes. The study populations are also heterogeneous, with some studies including all adult patients, and others focusing exclusively on older populations [24, 29, 41, 42] (most commonly those over 65 years of age). Additional sources of heterogeneity included country-level differences, variations in study quality and types of procedures included in each study (see Table 1 for characteristics of individual studies). The inclusion and exclusion criteria were designed to minimise heterogeneity, and a random-effects model was used in the meta-analysis to account for any remaining differences between studies. However, residual heterogeneity remained significant. Subgroup analysis was performed to stratify single-centre vs. multicentre data, study quality and retrospective vs. prospective design. Although no significant differences between groups were found, these findings must be interpreted with caution due to the small number of studies in each subgroup.

Given this degree of heterogeneity, the pooled estimates presented here represent system-level descriptive benchmarks rather than precise risk estimates for adverse outcomes. While these estimates may help inform risks observed in ambulatory surgery, they should not be applied as definitive risk estimates for specific surgical procedures or patient groups. Further research using standardised patient groups and outcome definitions is needed to generate accurate risk estimates for ambulatory surgical outcomes.

Secondly, the literature search was limited to PubMed and EMBASE databases. While these databases cover the majority of biomedical literature, this search may have missed studies in other databases, regional journals, and grey literature such as conference proceedings, and may introduce publication and language biases. This may affect the comprehensiveness of the data and the generalisability of conclusions drawn. Further reviews performed on this topic may benefit from a broader search strategy.

Thirdly, as our systematic review focused on studies reporting facility-wide outcomes with significant heterogeneity, results should not be used to guide surgical decisions or patient selection at the individual procedural level. Further research is required to stratify outcomes based on individual surgical specialities, using standardised outcome definitions to minimise heterogeneity.

Finally, only two-thirds of the included studies were rated as good quality, which limits the conclusions drawn from the results.

## Conclusions

Despite the proliferation of ambulatory surgery, relatively few studies have reported the outcomes across the spectrum of procedures performed in this setting. Ambulatory surgery appears to be associated with a relatively low conversion rate to inpatient hospitalisation, although this is often due to potentially preventable reasons. However, post-discharge outcomes have not been widely reported. Moreover, the marked heterogeneity in the cohort and outcome definitions suggests that these findings should be interpreted with caution and that the quality of ambulatory care remains uncertain. Further high-quality studies are needed to determine the quality of ambulatory surgery. 

## Supplementary Information

Supplementary Material 1.

## Data Availability

No datasets were generated or analysed during the current study.
