# Supplementary material for: Quality of outpatient ambulatory surgical care: a systematic review and meta-analysis
Source: BMC Surg. 2026 Apr 21;26:397. doi: 10.1186/s12893-026-03737-y (PMC13251288; doi:10.1186/s12893-026-03737-y)

**Supplementary Material**

**Quality of Ambulatory Surgical Care: A Systematic Review and Meta-Analysis**

**Authors**

Pooja Arumugam, Claire Fraser, Lisa Cassaniti, Joshua Wright, Sunnya Khawaja, Trang Dang, Alka Kothari, Manju Chandrasegaram, Isuru Ranasinghe.

### **Corresponding author**

Ms Pooja Arumugam, Northside Clinical Unit, Medical School, Faculty of Health, Medicine and Behavioural Sciences, Chermside, QLD 4027; email: [poojaarumugam68@gmail.com](mailto:poojaarumugam68@gmail.com)

### **Supplementary Figures and Tables**

Table S1: Literature Search Queries

Table S2: Summary of Individual Studies

Table S3: Quality Assessment of Individual Studies

Table S4: Comparison of results with arcsine and logit transformation

Table S5: PRISMA 2020 Checklist

Table S6: PRISMA Abstract Checklist

Figure S1: Funnel plot of the meta-analysis of immediate hospitalisation

Figure S2: Funnel plot of the meta-analysis of 30-day hospitalisation

**Table S1: Literature Search Queries**

| Database | Search Query |
| --- | --- |
| Pubmed | ("outpatient surgery"[tiab] OR "outpatient procedures" [tiab] OR "ambulatory surgery"[tiab] OR "ambulatory procedures"[tiab] OR "ambulatory care"[tiab] OR "day surgery"[tiab] OR "day case"[tiab] OR "day procedures"[tiab] OR "Ambulatory Surgical Procedures"[MeSH]) AND (“readmission*”[tiab] OR “rehospitalization*”[tiab] OR “return hospital visit*” [tiab] OR “complications” [tiab] OR “morbidity” [tiab] OR ((“unplanned”[tiab] OR “unanticipated” [tiab] OR “unexpected”[tiab]) AND (“admission*” [tiab] OR “hospitalization”[tiab])) OR "Patient Readmission"[MeSH]) NOT ("case reports"[Publication Type] OR "clinical conference"[Publication Type] OR "randomized controlled trial"[Publication Type])) AND (humans[Filter]) AND (alladult[Filter]) AND (English[Filter]) AND 2000/01/01:2023/12/31[Date - Publication]) |
| Embase | ('outpatient surgery':ti,ab OR 'outpatient procedures':ti,ab OR 'ambulatory surgery':ti,ab OR 'ambulatory procedures':ti,ab OR 'ambulatory care':ti,ab OR 'day surgery':ti,ab OR 'day case':ti,ab OR 'day procedures':ti,ab OR 'ambulatory surgical procedures'/exp) AND (readmission*:ti,ab OR rehospitalization*:ti,ab OR 'return hospital visit*':ti,ab OR complications:ti,ab OR morbidity:ti,ab OR ((unplanned:ti,ab OR unanticipated:ti,ab OR unexpected:ti,ab) AND (admission*:ti,ab OR hospitalization:ti,ab)) OR 'patient readmission'/exp) AND [01-01-2000 to 31-12-2023]/pd NOT ('conference abstract'/it OR 'review'/it) AND [english]/lim AND [adult]/lim |

**Table S2: Summary of Individual Studies**

| **Author** | **Year Published** | **Study Design** | **Country** | **Hospital/Database** | **Population Definition** | **Exclusion criteria for population** | **Time range of data collection** | **Outcome/s** | **Data source of outcome** | **Follow-Up time** | **Types of surgery** | **Type of Anaesthetic** |
| --- | --- | --- | --- | --- | --- | --- | --- | --- | --- | --- | --- | --- |
| **Alder** | 2023 | Retrospective cohort | USA | ACS-NSQIP | adult patients in 9 major ACS-SQIP surgical specialties undergoing outpatient surgery | not in the 9 ACS SQIP surgical specialities, missing data, ASA class IV or V, preoperative diagnosis of sepsis or ventilator dependence, emergency operations | 2005-2018 | 30-day mortality | ACS-NSQIP | 30 days | general surgery, gynecology, neurosurgery, orthopedic, otolaryngology, plastic, thoracic, urology, and vascular surgery | Not stated |
| **Aldwinckle** | 2004 | Retrospective cohort | England | Torbay Hospital | All patients undergoing day surgery, over 70 years of age | Not mentioned | 2002-2004 | Readmission and postoperative complications | post-operative follow up phone call | immediate for readmission, 24hr for complications | General, urology, orthopaedic, ENT, gynaecology, plastics, dermatology, maxillo-facial, opthalmology, pain relief | general and local |
| **Arance Garcia** | 2015 | prospective cohort | Spain | Hospital Duques de Infantado (HDI), Seville | [adults scheduled for day surgery under general or regional anaesthesia with or without sedation](https://www.sciencedirect.com/topics/medicine-and-dentistry/regional-anesthesia) | surgery at the HDI after the preliminary pre-anaesthesia visit, and patients refusing to undergo MDS | 2009-2010 | postoperative complications, readmission within 24hrs | Hospital Follow-up Data, post-op telephone call | immediate for readmissions, 24-48hyrs for complications | General, Orthopaedic, ENT and Urology surgery | Regional and general |
| **Awan** | 2013 | Retrospective cohort | Ireland | St James Hospital | ASA status I to III, between 18 to 85 years old, procedures lasting less than 90 minutes that are not expected to cause excessive fluid shift or physiological impairment postoperatively | not stated | 2011-2012 | Hospital admission, perioperative complications | Hospital Data | immediate | general surgery including breast vascular and GI | Not stated |
| **Bongiovanni** | 2021 | Retrospective cohort | USA | (AHRQ) Healthcare Cost and Utilization Project State Ambulatory Surgery and Services Databases (SASD), California data | All individuals who underwent same-day surgeries performed in HOPDs and ASCs | disposition was listed as missing, death, or left against medical advice, facilities that performed less than 100 surgeries | 2009-2011 | Unplanned hospital visits within 30 days | (AHRQ) Healthcare Cost and Utilization Project State Ambulatory Surgery and Services Databases (SASD), California data | 30-days | Muscular-skeletal, digestive system, integumentar, cardiovascular, urinary, male genitalia, female genitalia, nervous system | Not stated |
| **Cabaton** | 2021 | Retrospective cohort | France | Hoˆpital Prive´ Jean Mermoz, RamsayGe´ne´ rale de Sante´, Lyon, France | Every patient scheduled for ambulatory surgery | procedures performed under local anaesthesia without the assistance of an anaesthesiologist | 2015-2017 | hospital admission on the day of the procedure | Hospital Database | Day of procedure | Gastrointestinal endoscopy, urology, ENT, orthopaedic (lower limb, hand, shoulder), third molar extraction, visceral, breast, varicose vein, opthalmology, plastics, bronchial, other | Not stated |
| **Desai** | 2022 | Restrospective cohort | USA | Medicare and CMS data | 65 years and older, undergoing outpatient surgery, those that Medicare considers ‘‘substantive’’ based on a Medicare Physician Fee Schedule global surgery indicator (GSI) value of 090 | patients who lack continuous Medicare enrollment in the 7 days after surgery, same day acute hospital visits | 2015-2016 | unplanned hospital visit within 7 days of the outpatient surgery | Inpatient and outpatient institutional claims capturing hospital visits | 7 days | Any outpatient | Not stated |
| **Doan** | 2019 | Retrospective cross-sectional | USA | institutional electronic medical record (EMR; Epic, Verona, WI, USA | adult patients who underwent elective noncardiac surgery | cancelled on the day of surgery, erroneous data, patients who died in the hospital, cardiac, emergency and pediatric cases, patients on short acting opioids | 2011-2014 | hospital readmission, respiratory failure, adverse cardiac events, and, for inpatient surgeries, hospital length of stay | Hospital data, ICD codes | 30 days | All elective non cardiac | Not stated |
| **Dyas** | 2022 | Retrospective cohort | USA | ACS NSQIP | Adult outpatients | Outside of the 9 ACS SQIP surgical specialities, emergency patients, had sepsis or septic shock, ventilator dependence, American Society of Anesthesiologists (ASA) class IV or V, missing data | 2005-2018 | Conversion from outpatient to inpatient, post-op complications, readmissions, mortality | ACS NSQIP | 30 days | general surgery, gynecology, neurosurgery, orthopedics, otolaryngology, plastic, thoracic, urology, and vascular | Not stated |
| **Fahmy** | 2016 | Prospective cohort | London | Multisite trust in East London (name not specified) | Adult day case surgeries under general, regional or local anaesthetic, Patient consent to be contacted at home, Competent in English language without need for translator | Lack of patient consent, complication requiring patient admission to hospital | 2013 | Postoperative pain | post-op follow up phone call | 48 hours | Breast, General, Gynaecology, Orthopaedic, Urology and ENT surgery | general, regional, local |
| **Fox** | 2014 | Restrospective cohort | USA | (AHRQ) Healthcare Cost and Utilization Project (HCUP), California (CA), Florida (FL), and Nebraska (NE) ambulatory surgery, | All discharges for medical and surgical procedures, at least 18 years of age and had valid encrypted person-level identifiers | disposition was listed as missing, death, or left against medical advice | 2008-2009 | Rates of hospital transfer and hospital-based, acute care within 7 days of discharge | state-level inpatient and emergency department databases | 7 days | Any outpatient | Not stated |
| **Jiang** | 2021 | Retrospective cohort | China | West China Hospital | Elective day surgeries | Not specified | 2020 | fever (T ≥ 37.3°C), wound pain, nausea, vomiting, or other complications | Telephone follow-up | days 2,7, 14 and 30 after discharge | ultrasound-guided Mammotome biopsy, endoscopic polypectomy, hernia repair, pediatric surgeries, cholecystectomy, choledochoscopy, radical mastectomy, percutaneous transhepatic cholangial drainage, liver biopsy, video-assisted thoracoscopic surgery, laparoscopic resection of colon cancer, colostomy apotheosis, and varicose vein surgery | general, local, none |
| **Leardi** | 2008 | Retrospective cohort | Italy | Pescina hospital | All patients undergoing day surgery, over 18 years of age | Not mentioned | 2001-2007 | early readmission and Morbidity | Hospital Data | day 1,3,7 and beyond | Any outpatient | Local, arachnoid, truncular, general |
| **Lerman** | 2019 | Retrospective cohort | USA | VASQIP | adult patients undergoing ambulatory, elective, noncardiac surgery | Cardiac operations, emergency operations, and nonsurgical procedures (ie, bronchoscopy, pacemaker insertion, and endoscopy) | 2009-2016 | 30-day complication rate | VASQIP | 30 days | Any outpatient | General, epidural/spinal, monitored anesthesia care, local, other |
| **McIsaac** | 2015 | Retrospective cohort | Canada | Institute of Clinical Evaluative Sciences data | All Ontario residents aged ≥ 40 yr who underwent knee surgery, shoulder surgery, inguinal or femoral hernia repair, cholecystectomy, lumpectomy, or transurethral resection of urinary obstruction | patients less than 40, weekend surgeries | 2002-2012 | hospital readmission or an ED visit within 30 days of successful discharge following ambulatory surgery | Hospital record | 30 days | knee surgery, shoulder surgery, inguinal or femoral hernia repair, cholecystectomy, lumpectomy, or transurethral resection of urinary obstruction | Not stated |
| **Melton** | 2021 | Retrospective cohort | USA | MEDNAX, Inc. | adult patients (≥ 18 yr) undergoing procedures | missing anesthesia record, age < 18 yr or missing, ASA physical status > IV or missing, procedure classified as emergency, and missing or unidentified CPT codes | 2010-2014 | unplanned hospital admission within 24 hrs | MEDNAX, Inc. | 24 hrs | digestive, genitourinary, musculoskeletal, respiratory, or other | Not stated |
| **Menachemi** | 2008 | Retrospective cohort | USA | Florida Agency for Health Care Administration (AHCA) | Greater than 18 years undergoing ambulatory surgery | not specified | 2004 | Unexpected hospitalisation following surgery | Florida Agency for Health Care Administration (AHCA) | 7 and 30 days | colonoscopy, cataract removal, upper gastrointestinal endoscopy, arthroscopy, and biopsy of the prostate | Not stated |
| **Mihailescu** | 2020 | Prospective cross-sectional | France | Rouen University Hospital | adult patients scheduled for ambulatory surgery | Less than 18 years and patient refusal | 2017 | conversion to conventional hospitalisation or to emergency department visit within 30 days after discharge | Patient Records | 30 days | Ophthalmology, Gynaecology, Orthopaedic, Plastic, Ear-nose-throat, Maxillofacial, Hand surgery, Vascular, Arteriography, Pneumology, Odontology, Sismotherapy, General surgery (cholecystectomy, umbilical hernia, endometriosis, anal fistula) | general, local, locoregional |
| **Mull** | 2019 | Retrospective cohort | USA | Veterans Health Administration (VA) and Medicare | CMS and VA dually-enrolled patients over age 65 | not specified | 2011-2013 | ED visits | VA Information Resource Center’s VA/CMS data | 7 days | general, urology, orthopedic, ear/nose/throat and podiatry | Not stated |
| **Mull** | 2018 | Retrospective cohort | USA | VA CDW, VA NSO, AHRF, CMS | CMS and VA dually enrolled patients aged 65 and older, outpatient surgeries | inpatient surgery, under age 65, not one of 5 surgical specialities, emergency surgeries, ASA greater than V | 2011-2013 | postoperative outcomes and admissions | Hospital Data | 7 days, 14 days | general surgery, orthopedics, urology, ear/nose/throat, and podiatry | Not stated |
| **Odonkor** | 2015 | Prospective longitudinal | USA | 966-bed academic teaching hospital (name not specified) | adult patients (≥18 yrs) undergoing same-day surgery without planned or scheduled revisits | emergency surgeries, follow-up procedures, leaving against medical advice; 24-hr observation stays; same-day transfer to psychiatric facility, intensive care unit, or medical floor; oncology ward transfer; transfer to other hospitals; inpatient rehabilitation; alcohol and drug treatment; labor and delivery admissions; and mortality during index procedure. Repeat procedures and cancelled procedures | 2011-2012 | 30 day readmissions , ED visits | Hospital data | 30 days | arthroscopy, appendectomy, cholecystectomy, lump ectomy, cataract surgery, thyroid/parathyroidectomy, hernia surgery, polypectomy, and wound dehiscence | Not stated |
| **Odonkor** | 2013 | Prospective cohort | USA | Academic teaching hospital (name not specified) | adult patients (≥18 yrs) undergoing elective ambulatory surgical procedures at a major academic hospital center | back pain and movement disorders and those with anticipated lower limb surgery or the inability to complete the gait speed test | not specified | readiness for home discharge in ≤90 mins, unplanned admissions, postop complications | Hospital Data | Not mentioned for readmission, 24 hrs for complications | arthroscopy, bladder and kidney, upper and lower GI, lens and cateract, skin wound debridement, septoplasty, tympanoplasy | Not stated |
| **Owens** | 2014 | Retrospective cohort | USA | AHRQ 2010 data | low- to moderate-risk surgical procedures performed in hospital-owned ambulatory surgery settings | Jan or Dec surgeries, length of stay greater than 2 days, more than 1 surgery on same day, previous surgery in last 30 days, younger than 18 years | 2010 | clinically significant surgical site infections requiring admission, other caues of postsurgical hospital visits | ICD-9-CM diagnosis codes, ICD-9-CM or CPT procedure codes | 14 and 30 days | laparoscopic cholecystectomy, hernia repair, orthopedic, neurosurgery, gynaecology, urological | Not stated |
| **Peuchot** | 2020 | Retrospective before/after | France | French university hospital center (name not specified) | Patients over 18 scheduled for outpatient surgery with a mobile phone | pregnant, younger than age 18 years, or did not speak French | 2016-2017 | hospitalisations, post-op complications | Hospital data | 1 and 7 days | orthopedic; ear, nose, and throat; oral; dental; gynecologic; vascular; and thoracic | general, regional, spinal |
| **Pfisterer** | 2001 | prospective cohort | International (Czech Republic, Egypt, Estonia, Germany, Iceland, Italy, Norway, New Zealand, UK) | Not mentioned | over 18 years of age, underwent, surgery that did not require hospitalisation or a stay of more than 24 h in the day-case unit | illiterate, mentally impaired or unable to follow instructions | Not mentioned | PONV and Pain | Patient diary card | 4 days | general, obstetrics and gynaecology, ortho, GIT | Not stated |
| **Sawhney** | 2020 | Retrospective cohort | Canada | Institute of Clinical Evaluative Sciences Data | adults aged 18 years and older residing in Ontario who underwent select ambulatory surgical procedures | ED visit immediately prior to their surgery, death on day of surgery, no Ontario health insurance coverage, death on day of surgery | 2014-2018 | ED visit and hopsital admissions | NACRS or DAD | 3 days | hernia-related muscle repair of the chest and abdomen, cholecystectomy, knee joint repair, release of nerves in the forearm, shoulder surgery, tonsillectomy, tympanic membrane procedures, appendectomy and partial hysterectomy | Not stated |
| **Scott** | 2018 | Prospective cohort | UK | St. Mary’s Hospital | adult patients undergoing elective day-case general surgical procedures | bariatric, emergency or minor dermatological procedures and those with a urinary catheter in situ prior to, or inserted during, the procedure | 2014 | Postoperative urinary retention | Local emergency department data | 24 hrs | perianal procedures (e.g. examination of the anorectum under anaesthesia and treatment of fistulas, fissure-in-ano, haemorrhoidal disease and warts), laparoscopic procedures (e.g. laparoscopic hernia repair, cholecystectomy and fundoplication) and open hernia repair (e.g. inguinal and ventral hernioplasty | Not stated |
| **Stessel** | 2015 | Prospective cohort | Netherlands | Maastricht University Medical Center | All patients undergoing day surgery | patients age <18 years, inability to express themselves, visual dysfunction, or insufficient understanding of the Dutch language | 2008-2010 | QOR, GSR and QOL. Pre- and postoperative pain, visits to healthcare professionals, unplanned admissions and readmissions, postoperative complications | QOR questionnaires | 4 days | Any Outpatient | general, loco-regional |
| **Teja** | 2020 | Retrospective cohort | USA | Massachusetts General Hospital (MGH), Beth Israel Deaconess Medical Center (BIDMC), state of New York | adult patients undergoing ambulatory procedures under anesthesia care | Patients ≤17 years of age and patients undergoing cardiac procedures | 2007-2017 | 30 day unplanned admissions | ICD Codes | 30 days | Any outpatient | Not stated |
| **Van Caelenberg** | 2019 | Retrospective cohort | Belgium | University Hospital of Ghent | adults(18 years or older) planned for ambulatory surgery | Pediatric, ambulatory procedures per-formed outside the operating theatre (gastroscopy,colonoscopy, extracorporeal shock waves litho-tripsy and non-operating room procedural sedation) | 2016 | incidence, reasons and risk factors for unanticipated admission | hospital’s Electronic Patients Database (EPD | Immediate | Any outpatient | Not stated |

**Table S3: Quality Assessment of Individual Studies**

| Question | 1 | 2 | 3 | 4 | 5 | 6 | 7 | 8 | 9 | 10 | 11 | 12 | 13 | 14 | Rating |
| --- | --- | --- | --- | --- | --- | --- | --- | --- | --- | --- | --- | --- | --- | --- | --- |
| Alder | YES | YES | YES | YES | NA | YES | YES | NA | YES | NA | YES | NA | YES | NA | **GOOD** |
| Aldwinckle | YES | YES | YES | YES | NA | YES | YES | NA | YES | NA | YES | NA | YES | NA | **GOOD** |
| Arance Garcia | YES | YES | YES | YES | NA | YES | YES | NA | YES | NA | YES | NA | YES | NA | **GOOD** |
| Awan | YES | YES | YES | YES | NA | YES | YES | NA | YES | NA | YES | NA | YES | NA | **GOOD** |
| Bongiovanni | YES | YES | YES | YES | NA | YES | YES | NA | YES | NA | YES | NA | YES | NA | **GOOD** |
| Cabaton | YES | YES | YES | YES | NA | YES | YES | NA | YES | NA | YES | NA | YES | NA | **GOOD** |
| Desai | YES | YES | YES | YES | NA | YES | YES | NA | YES | NA | YES | NA | YES | NA | **GOOD** |
| Doan | YES | YES | YES | YES | NA | YES | YES | NA | YES | NA | YES | NA | YES | NA | **GOOD** |
| Dyas | YES | YES | YES | YES | NA | YES | YES | NA | YES | NA | YES | NA | YES | NA | **GOOD** |
| Fahmy | YES | NO | YES | YES | NA | YES | YES | NA | YES | NA | YES | NA | NO | NA | **FAIR** |
| Fox | YES | YES | YES | YES | NA | YES | YES | NA | YES | NA | YES | NA | YES | NA | **GOOD** |
| Jiang | YES | NO | Unsure | YES | NA | YES | YES | NA | YES | NA | NO | NA | YES | NA | **FAIR** |
| Leardi | YES | YES | YES | YES | NA | YES | YES | NA | YES | NA | NO | NA | YES | NA | **FAIR** |
| Lerman | YES | YES | YES | YES | NA | YES | YES | NA | YES | NA | YES | NA | YES | NA | **GOOD** |
| McIsaac | YES | YES | YES | YES | NA | YES | YES | NA | YES | NA | YES | NA | YES | NA | **GOOD** |
| Melton | YES | YES | YES | YES | NA | YES | YES | NA | YES | NA | YES | NA | YES | NA | **GOOD** |
| Menachemi | YES | YES | YES | YES | NA | YES | YES | NA | YES | NA | YES | NA | YES | NA | **GOOD** |
| Mihailescu | YES | YES | YES | YES | YES | YES | YES | NA | YES | NA | YES | NA | YES | NA | **GOOD** |
| Mull | YES | YES | YES | YES | NA | YES | YES | NA | YES | NA | YES | NA | YES | NA | **GOOD** |
| Mull | YES | YES | YES | YES | NA | YES | YES | NA | YES | NA | NO | NA | YES | NA | **FAIR** |
| Odonkor | YES | YES | YES | YES | NA | YES | YES | NA | YES | NA | YES | NA | YES | NA | **FAIR** |
| Odonkor | YES | YES | YES | YES | YES | YES | Unsure | NA | NO | NA | NO | NA | NO | NA | **POOR** |
| Owens | YES | YES | YES | YES | NA | YES | YES | NA | YES | NA | YES | NA | YES | NA | **GOOD** |
| Peuchot | YES | YES | YES | YES | NA | YES | YES | NA | YES | NA | NO | NA | YES | NA | **FAIR** |
| Pfisterer | YES | NO | Unsure | YES | NO | YES | YES | NA | YES | NA | YES | NA | YES | NA | **FAIR** |
| Sawhney | YES | YES | YES | YES | NA | YES | YES | NA | YES | NA | YES | NA | YES | NA | **GOOD** |
| Scott | YES | YES | YES | YES | NA | YES | YES | NA | YES | NA | YES | NA | YES | NA | **GOOD** |
| Stessel | YES | YES | NO | YES | NA | YES | YES | NA | YES | NA | YES | NA | YES | NA | **FAIR** |
| Teja | YES | YES | YES | YES | NA | YES | YES | NA | YES | NA | YES | NA | YES | NA | **GOOD** |
| Van Caelenberg | YES | YES | YES | YES | NA | YES | YES | NA | YES | NA | NO | NA | YES | NA | **FAIR** |

**Table S4: Comparison of results with arcsine and logit transformation**

| Outcome | Pooled Proportion (95% CI) (%) | |
| --- | --- | --- |
|  | **Arcsine** | **Logit** |
| Immediate Readmissions - Total | 1.82 (1.10-2.71) | 1.46 (0.80-2.67) |
| 30-Day Readmissions | 2.88 (1.20-5.26) | 2.43 (1.43-4.08) |
| Immediate - good studies | 1.81 (0.94-2.95) | 1.39 (0.64-2.98) |
| Immediate - fair studies | 1.86 (0.91-3.13) | 1.74 (0.91-3.31) |
| Immediate - individual hospitals | 2.00 (1.35-2.77) | 1.92 (1.33-2.77) |
| Immediate - Databases | 1.56 (0.43-3.36) | 1.01 (0.28-3.56) |
| Immediate - retrospective | 1.60 (0.89-2.51) | 1.26 (0.63-2.47) |
| Immediate - prospective | 3.18 (1.96, 4.67) | 3.17 (2.08-4.82) |

**Table S5: PRISMA 2020 Checklist**

| **Topic** | **No.** | **Item** | **Location where item is reported** |
| --- | --- | --- | --- |
| **TITLE** |  |  |  |
| **Title** | 1 | Identify the report as a systematic review. | Title |
| **ABSTRACT** |  |  |  |
| **Abstract** | 2 | See the PRISMA 2020 for Abstracts checklist |  |
| **INTRODUCTION** |  |  |  |
| **Rationale** | 3 | Describe the rationale for the review in the context of existing knowledge. | Introduction, page 3 |
| **Objectives** | 4 | Provide an explicit statement of the objective(s) or question(s) the review addresses. | Introduction, page 3+4 |
| **METHODS** |  |  |  |
| **Eligibility criteria** | 5 | Specify the inclusion and exclusion criteria for the review and how studies were grouped for the syntheses. | Methods, page 4 |
| **Information sources** | 6 | Specify all databases, registers, websites, organisations, reference lists and other sources searched or consulted to identify studies. Specify the date when each source was last searched or consulted. | Methods, page 4 |
| **Search strategy** | 7 | Present the full search strategies for all databases, registers and websites, including any filters and limits used. | Supplementary Materials, page 2 |
| **Selection process** | 8 | Specify the methods used to decide whether a study met the inclusion criteria of the review, including how many reviewers screened each record and each report retrieved, whether they worked independently, and if applicable, details of automation tools used in the process. | Methods, page 4+5 |
| **Data collection process** | 9 | Specify the methods used to collect data from reports, including how many reviewers collected data from each report, whether they worked independently, any processes for obtaining or confirming data from study investigators, and if applicable, details of automation tools used in the process. | Methods, page 5+6 |
| **Data items** | 10a | List and define all outcomes for which data were sought. Specify whether all results that were compatible with each outcome domain in each study were sought (e.g. for all measures, time points, analyses), and if not, the methods used to decide which results to collect. | Methods, page 5 |
|  | 10b | List and define all other variables for which data were sought (e.g. participant and intervention characteristics, funding sources). Describe any assumptions made about any missing or unclear information. | Supplementary Materials, page 3-12 |
| **Study risk of bias assessment** | 11 | Specify the methods used to assess risk of bias in the included studies, including details of the tool(s) used, how many reviewers assessed each study and whether they worked independently, and if applicable, details of automation tools used in the process. | Methods, page 6 |
| **Effect measures** | 12 | Specify for each outcome the effect measure(s) (e.g. risk ratio, mean difference) used in the synthesis or presentation of results. | Methods, page 6 |
| **Synthesis methods** | 13a | Describe the processes used to decide which studies were eligible for each synthesis (e.g. tabulating the study intervention characteristics and comparing against the planned groups for each synthesis (item 5)). | Methods, page 5 |
|  | 13b | Describe any methods required to prepare the data for presentation or synthesis, such as handling of missing summary statistics, or data conversions. | Methods, page 5 |
|  | 13c | Describe any methods used to tabulate or visually display results of individual studies and syntheses. | Methods, page 6 |
|  | 13d | Describe any methods used to synthesize results and provide a rationale for the choice(s). If meta-analysis was performed, describe the model(s), method(s) to identify the presence and extent of statistical heterogeneity, and software package(s) used. | Methods, page 6 |
|  | 13e | Describe any methods used to explore possible causes of heterogeneity among study results (e.g. subgroup analysis, meta-regression). | Methods, page 6 |
|  | 13f | Describe any sensitivity analyses conducted to assess robustness of the synthesized results. | Methods, page 6 |
| **Reporting bias assessment** | 14 | Describe any methods used to assess risk of bias due to missing results in a synthesis (arising from reporting biases). | Methods, page 6 |
| **Certainty assessment** | 15 | Describe any methods used to assess certainty (or confidence) in the body of evidence for an outcome. | Not Reported |
| **RESULTS** |  |  |  |
| **Study selection** | 16a | Describe the results of the search and selection process, from the number of records identified in the search to the number of studies included in the review, ideally using a flow diagram. | Results, page 8-10; Figure 1 PRISMA flow diagram |
|  | 16b | Cite studies that might appear to meet the inclusion criteria, but which were excluded, and explain why they were excluded. | Not Reported |
| **Study characteristics** | 17 | Cite each included study and present its characteristics. | Supplementary Materials, page 3-12 |
| **Risk of bias in studies** | 18 | Present assessments of risk of bias for each included study. | Supplementary Materials, page 23-24  Results page 17 |
| **Results of individual studies** | 19 | For all outcomes, present, for each study: (a) summary statistics for each group (where appropriate) and (b) an effect estimate and its precision (e.g. confidence/credible interval), ideally using structured tables or plots. | Figures 2-5 |
| **Results of syntheses** | 20a | For each synthesis, briefly summarise the characteristics and risk of bias among contributing studies. | Results page 10-17  Supplementary Materials, page 23-24 |
|  | 20b | Present results of all statistical syntheses conducted. If meta-analysis was done, present for each the summary estimate and its precision (e.g. confidence/credible interval) and measures of statistical heterogeneity. If comparing groups, describe the direction of the effect. | Results page 10-18; Figures 2-5 |
|  | 20c | Present results of all investigations of possible causes of heterogeneity among study results. | Results, page 18 |
|  | 20d | Present results of all sensitivity analyses conducted to assess the robustness of the synthesized results. | Supplementary materials, page 15, |
| **Reporting biases** | 21 | Present assessments of risk of bias due to missing results (arising from reporting biases) for each synthesis assessed. | Results, page 23,24 |
| **Certainty of evidence** | 22 | Present assessments of certainty (or confidence) in the body of evidence for each outcome assessed. | Not Reported |
| **DISCUSSION** |  |  |  |
| **Discussion** | 23a | Provide a general interpretation of the results in the context of other evidence. | Discussion, page 18-19 |
|  | 23b | Discuss any limitations of the evidence included in the review. | Discussion, page 22-23 |
|  | 23c | Discuss any limitations of the review processes used. | Discussion, page 22-23 |
|  | 23d | Discuss implications of the results for practice, policy, and future research. | Discussion, page 19-22 |
| **OTHER INFORMATION** |  |  |  |
| **Registration and protocol** | 24a | Provide registration information for the review, including register name and registration number, or state that the review was not registered. | Not Registered |
|  | 24b | Indicate where the review protocol can be accessed, or state that a protocol was not prepared. | Not Prepared |
|  | 24c | Describe and explain any amendments to information provided at registration or in the protocol. | NA |
| **Support** | 25 | Describe sources of financial or non-financial support for the review, and the role of the funders or sponsors in the review. | NA |
| **Competing interests** | 26 | Declare any competing interests of review authors. | No competing interests |
| **Availability of data, code and other materials** | 27 | Report which of the following are publicly available and where they can be found: template data collection forms; data extracted from included studies; data used for all analyses; analytic code; any other materials used in the review. | Not Available |

#####

**Table S6: PRISMA Abstract Checklist**

| **Topic** | **No.** | **Item** | **Reported?** |
| --- | --- | --- | --- |
| **TITLE** |  |  |  |
| **Title** | 1 | Identify the report as a systematic review. | Yes |
| **BACKGROUND** |  |  |  |
| **Objectives** | 2 | Provide an explicit statement of the main objective(s) or question(s) the review addresses. | Yes |
| **METHODS** |  |  |  |
| **Eligibility criteria** | 3 | Specify the inclusion and exclusion criteria for the review. | Yes |
| **Information sources** | 4 | Specify the information sources (e.g. databases, registers) used to identify studies and the date when each was last searched. | Yes |
| **Risk of bias** | 5 | Specify the methods used to assess risk of bias in the included studies. | Yes |
| **Synthesis of results** | 6 | Specify the methods used to present and synthesize results. | Yes |
| **RESULTS** |  |  |  |
| **Included studies** | 7 | Give the total number of included studies and participants and summarise relevant characteristics of studies. | Yes |
| **Synthesis of results** | 8 | Present results for main outcomes, preferably indicating the number of included studies and participants for each. If meta-analysis was done, report the summary estimate and confidence/credible interval. If comparing groups, indicate the direction of the effect (i.e. which group is favoured). | Yes |
| **DISCUSSION** |  |  |  |
| **Limitations of evidence** | 9 | Provide a brief summary of the limitations of the evidence included in the review (e.g. study risk of bias, inconsistency and imprecision). | Yes |
| **Interpretation** | 10 | Provide a general interpretation of the results and important implications. | Yes |
| **OTHER** |  |  |  |
| **Funding** | 11 | Specify the primary source of funding for the review. | Yes |
| **Registration** | 12 | Provide the register name and registration number. | No |

**Figure S1: Funnel plot of the meta-analysis of immediate hospitalisation**


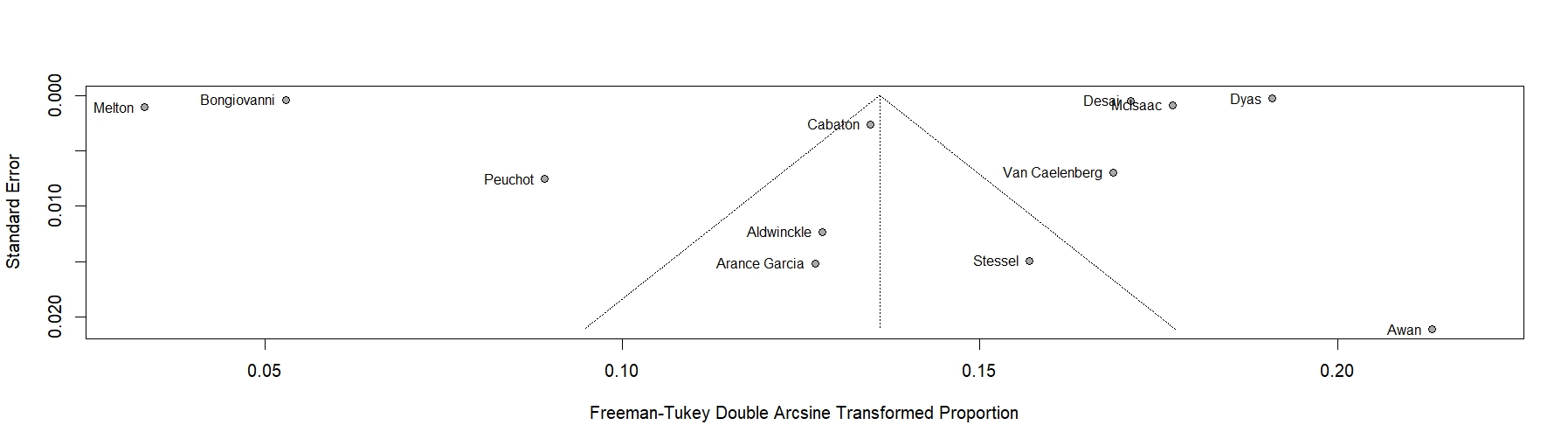


**Figure S2: Funnel plot of the meta-analysis of 30-day hospitalisation**


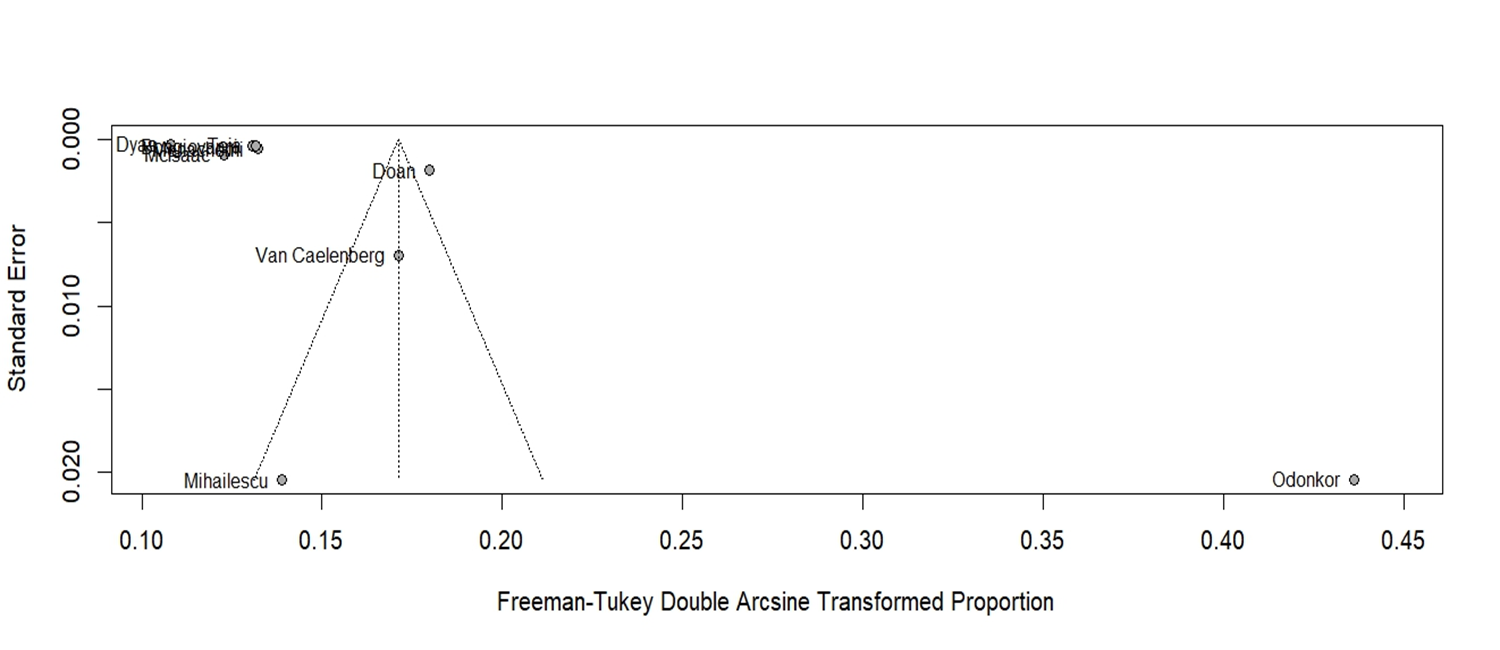

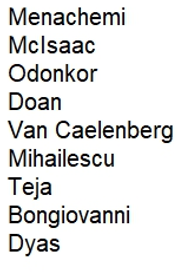

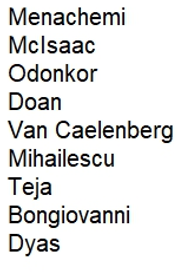

Supplement: Supplementary file 1 — Supplementary Material 1. [file 12893_2026_3737_MOESM1_ESM.docx]
